# Evaluating the referral preferences and consultation requests of primary care physicians with otolaryngology – head and neck surgery

**DOI:** 10.1186/s40463-015-0114-2

**Published:** 2015-12-29

**Authors:** John R. Scott, Eric Wong, Leigh J Sowerby

**Affiliations:** Department of Otolaryngology – Head and Neck Surgery, Schulich School of Medicine and Dentistry, Western University, St. Joseph’s Healthcare, 268 Grosvenor Street, London, ON N6A 4 V2 Canada; Department of Family Medicine, Schulich School of Medicine and Dentistry, Western University, London, Ontario Canada

**Keywords:** Referral, Consultation, Otolaryngology, Continuity of care, Central referral system, General practitioners, Primary care providers, Family medicine

## Abstract

**Background:**

No literature exists which examines referral preferences to, or the consultation process with, Otolaryngology. In a recent Canadian Medical Association nation-wide survey of General Practitioners and Family Physicians, Otolaryngology was listed as the second-most problematic specialty for referrals. The purpose of this study was to learn about and improve upon the referral process between primary care physicians (PCPs) and Otolaryngology at an academic centre in Southwestern Ontario.

**Methods:**

PCPs who actively refer patients to Otolaryngology within the catchment area of Western University were asked to complete a short paper-based questionnaire. Data was analyzed using descriptive statistics.

**Results:**

A total of 50 PCPs were surveyed. Subspecialty influenced 90.0 % of the referrals made. Specialist wait times altered 58.0 % of referrals. All PCPs preferred to communicate via fax. Half of those surveyed wanted clinical notes from every encounter. Seventy-four percent of respondents wanted inappropriate referrals forwarded to the proper specialist automatically. Twenty-two percent of those surveyed were satisfied with current wait times. A central referral system was favored by 74 % of PCPs.

**Conclusion:**

Improvements could help streamline the referral and consultation practices with Otolaryngology in Southwestern Ontario. A central referral system and reduction in the frequency of consultative reports can be considered.

**Electronic supplementary material:**

The online version of this article (doi:10.1186/s40463-015-0114-2) contains supplementary material, which is available to authorized users.

## Background

In Canada, specialists rely heavily upon primary care physicians (PCPs) as the main source for their referral base. Although variable among provinces and hospitals, the process typically begins with a PCP requesting specialty consultation [1]. Research exists that attempted to improve the quality of referrals; however, the results are largely equivocal and the studies were not specific to any one specialty [2]. Since the referral and consultation process is critical to facilitating timely access to care it is essential that both aspects are evaluated [3].

Recently, the Canadian Medical Association (CMA) has looked into addressing this issue by performing a serial survey of General Practitioners/Family Physicians on the topic. The results of their research have not been published but some of the findings are posted on their website (www.cma.ca/referrals). When it came to referral requests by General Practitioners/Family Physicians to specialists, preferences seemed to vary widely according to the CMA survey. Furthermore, Otolaryngology was listed as the second-most problematic specialty for referrals.

To date, there is no literature specifically studying referral preferences to Otolaryngology from PCPs. In addition, there is no literature examining the consultation requests of PCPs when referring to Otolaryngology. For these reasons, and because of the results of the CMA survey we endeavored to evaluate the service provided by our department. The specific objectives for this study are two-fold. One, to gauge the consultation service provided by Otolaryngology in Southwestern Ontario, and two, to assess preferences and factors influencing PCP referrals to Otolaryngology.

## Methods

A cross-sectional survey was performed after Health Sciences Research Ethics Board approval was received from our institution (File No. 104575). Inclusion criteria were PCPs within the Western University catchment areas that have actively referred patients to Otolaryngology within the past year. There were no additional exclusion criteria. The questionnaire was paper-based and took approximately 10 min to complete. The survey was first piloted at the 2013 Family Medicine Otolaryngology Update Course at Western University. After appropriate modifications, the survey was faxed to all PCPs in the catchment area. Upon completion, all surveys were returned via fax. The majority of referrals to incorrect subspecialty Otolaryngologists were re-faxed back to the corresponding PCP and also onto the appropriate consultant at our center.

The questionnaire involved 32 questions and was sectioned into Demographics, Communication, Referral, Consultation and General Comments. Answer options were in the form of yes/no, multiple choice, 5-point Likert scale and free text. The actual survey distributed to PCPs is included (see Additional file 1). Surveys were faxed to PCPs. Data was evaluated using descriptive statistical analyses in Microsoft Office® Excel 2011.

## Results

A total of 210 surveys were sent out to local PCPs. Of these, 50 were returned and included in the study (23.8 % response rate). The average age of responders was 53.0 years (SD 11.8) and the majority was female. Two-thirds of PCPs practiced in an urban/suburban setting and most worked in a private group practice. Table 1 outlines the characteristics of the respondents.

**Table 1:** Characteristics of respondents

Characteristic	Number (percent)
Age in years (mean 53.0, SD 11.8)	
30 – 40	9 (18 %)
41 – 50	10 (20 %)
51 – 60	17 (34 %)
61 – 70	13 (26 %)
> 70	1 (2 %)
Female gender	27 (54 %)
Patients referred to otolaryngology per year	
< 10	5 (10 %)
10 – 20	16 (32 %)
21 – 40	17 (34 %)
41 – 60	4 (8 %)
61 – 80	6 (12 %)
> 80	2 (4 %)
Primary population served	
Inner City	9 (18 %)
Suburban/urban	33 (66 %)
Small Town	4 (8 %)
Rural	3 (6 %)
Geographically Remote	1 (2 %)
Primary patient care setting	
Private	33 (66 %)
Community Clinic	6 (12 %)
Walk-In-Clinic	4 (8 %)
Academic Health Sciences Center	4 (8 %)
Community Hospital	3 (6 %)
Practice Organization	
Solo	18 (36 %)
Group	30 (60 %)
Other	2 (4 %)

Electronic Medical Records (EMRs) were utilized by 74.0 % of those surveyed. In this group, 92.0 % used a standard referral template generated by the EMR. Only one of the 50 respondents had an Otolaryngology-specific template. All respondents preferred to communicate via fax as opposed to email, mailed letter or telephone. Seventy-eight percent of respondents ordered additional tests as they saw fit with their referrals. Regulations or restrictions that prevent PCPs from ordering advanced tests were only thought to create inefficiencies by 16.0 % of respondents.

The PCPs who responded in this study described wait times (58.0 %), personal relationships (68.0 %) and word of mouth from patients or colleagues (50.0 %) as playing a role in their decision to refer to a specific Otolaryngologist. Ninety percent of respondents indicated the largest influence on the choice of Otolaryngologist was the subspecialty of the problem. Interestingly, however, 66.0 % of respondents found it difficult to know who sees what types of particular problems in Otolaryngology. A central referral system was favored by 74.0 % of respondents and was also described in the free comment section.

Should an Otolaryngologist not deal with a certain problem, 76.0 % of respondents stated they would prefer the referral be forwarded automatically to the proper Otolaryngologist (see Table 2). With respect to receiving notes from Otolaryngology, respondents preferred them after the initial consultation, with a change in management, and when surgery has been performed. Only half of the respondents wanted notes from every clinical encounter. These findings are presented in Table 3.

**Table 2:** Respondent preference when an Otolaryngologist does not treat the referred problem

Action of the otolaryngologist	Percent of PCP’s that agreed
Referral sent back to PCP’s office	20 %
Different specialist recommended	44 %
Forward directly to the proper person	74 %

**Table 3:** Respondent preference on receiving notes from Otolaryngology

Clinical scenario	Percent of PCP’s that agreed
Initial consultation	94 %
Change in management	92 %
Operative note	86 %
Every clinical encounter	50 %

Overall, only 22.0 % of respondents were happy with current wait times at our centre; however, 88.0 % agreed that after initial consultation, care and communication were provided in an acceptable timeframe. Less than half (40.0 %) of respondents wanted the Otolaryngologist to remain highly involved in the care of their patient after recommendations were made regarding non-surgical issues. Fifty-four percent of respondents were satisfied with the referral process and 66.0 % were content with the existing consultation service in London. Themes of suggestion from the free text section included an annually updated list of subspecialty preferences, an urgent Otolaryngology clinic, and a central referral system.

## Discussion

The present study demonstrated that changes should be made to both the referral and consultation practices in Southwestern Ontario between PCPs and Otolaryngology. Despite a small sample size the respondent PCPs were similar in their gender ratio as compared to Southwestern Ontario PCPs overall. This was evident as 54.0 % were female as compared to 51.4 % of female PCPs practicing in the Southwestern Ontario PCP population [4]. In regards to the referral process, subspecialty had the greatest influence on PCPs decision-making, although most found it difficult to know who sees what particular problem. Individual wait times and personal relationships played a role as well but to a lesser extent. The majority of PCPs used EMR-generated referral templates and only one had an Otolaryngology-specific template. Somewhat surprisingly, wait times did not seem to play a major role in the referral algorithm of the respondents. This may stem from comparable wait times across staff Otolaryngologists in London. It is not infrequent that non-urgent consults wait upwards of a year to be seen. At our centre there is no specific Urgent Otolaryngology clinic but any consultant’s clinic can add on urgent referrals and thus they are triaged and seen accordingly.

With respect to the consultation practices, respondent PCPs stated a desire for inappropriate referrals to be forwarded automatically to the proper surgeon. They were divided in their preference for wanting notes from every encounter but do welcome them after the initial consultation, with a change in management and after surgery has been performed. Additionally, since all respondents preferred to communicate via fax, these results suggest that considerable cost savings can potentially be achieved if the resources associated with mailing appointment information or routine clinic follow-up notes are utilized elsewhere. Although the forwarding of inappropriate referrals to the correct specialist seems straightforward in theory, it can be a frustrating runaround for the PCP and Otolaryngologist alike. One possible solution to this includes providing local PCPs with a regularly updated list of subspecialist interests and preferences. Another option may involve a nurse-led triage system which was recently demonstrated to be effective and safe in a pediatric Otolaryngology referral setting [3]. Future work in this area will begin by considering some of the outlined changes requested by respondents. It would be advantageous to repeat the survey in a few years and see if improvements in the referral process have been realized. The central referral system suggestion was one made not only in this study but was a large part of the previous CMA survey.

The concept of a centralized system is not a new idea, nor is it something foreign to the health care system in Canada. In the United Kingdom, pooled specialist referrals have been in use for well over a decade [1]. Previously, a ‘Choose and Book’ method was attempted in England whereby PCPs triaged Otolaryngology referrals and they themselves booked their patients into consultant clinics [5]. This approach did not receive widespread support for several reasons, including a 270 % increase in urgent referrals, and has since fallen out of practice [6]. In more recent years, the province of Saskatchewan has made a push toward a centralized system. This system has benefits for patients, referring physicians and specialists. The goal of centralized systems is not to reduce patient wait times but rather to better direct patient flow and equally distribute workloads [7]. Patients may still wait to see a specific surgeon but they also have the option of seeing the first available specialist in a pool. It should be noted that such systems function best when multiple specialists are involved. When adopting pooled referrals the most significant time investment comes with the initial startup. Implementation guidelines do exist which has been complied by select provincial governments [7]. Unifying the referral process through centralization has the potential to address many of the concerns expressed by PCPs in this study.

The study presented here does have several limitations largely stemming from its small sample size and voluntary response bias. The response rate of roughly 1 in 4 may have been higher with an e-survey but given the preference for fax discovered in the pilot questionnaire we chose this form of distribution. In theory, there was also potential for frontline staff to discard the faxed survey as junk mail, which would have negatively affected our response rate. This paper focused exclusively on referrals to London-based Otolaryngologists and the opinions may not be generalizable outside our catchment area or to non-tertiary care referral centers. Nevertheless, the survey highlights the importance of a needs-based assessment in helping to optimize communication practices for patient care.

## Conclusion

In summary, results of our study support improvements in both the referral and consultation process between PCPs and Otolaryngology in Southwestern Ontario. A centralized referral system appears to be a referral process change that can address concerns expressed by our respondent PCPs. Such a system would need to be able to re-direct inappropriate referrals to the appropriate specialist. With respect to the consultative process, savings and efficiencies may be possible with consideration to reduce the number of progress reports to just the initial consultation, subsequent surgeries and significant changes in management.

## Additional file

Additional file 1:
**Primary care physician questionnaire. Questionnaire used in the study.** (PDF 63 kb)

## Abbreviations

PCP: Primary care physician
CMA: Canadian Medical Association
EMR: Electronic medical record
