# Supplementary material for: Evaluating the referral preferences and consultation requests of primary care physicians with otolaryngology – head and neck surgery
Source: J Otolaryngol Head Neck Surg. 2015 Dec 29;44:57. doi: 10.1186/s40463-015-0114-2 (PMC4696345; doi:10.1186/s40463-015-0114-2)
Supplement: Additional file 1: — Primary care physician questionnaire. Questionnaire used in the study. (PDF 63 kb) [file 40463_2015_114_MOESM1_ESM.pdf]

Referral Questionnaire for General Practitioners

The following questionnaire is voluntary. Data files generated from completed surveys will never be matched to individual names or addresses. Analysis and publication of survey results will be made will be made available publically.

Please circle an option or fill in the blank as required.

1. Are you currently a practicing General Practitioner or Family Physician in Canada?

Yes No

2. Do you actively refer patients to Otolaryngology?

Yes No

NOTE: These are exclusion questions. If you have answered "No" to either Question 1 or 2 please do not continue with the questionnaire.

3. How many patients do you typically refer to Otolaryngology per year?

<10 10 - 20 21 – 40 41-60 61-80 >80

DEMOGRAPHICS

4. What is your year of birth: _________

5. What is your gender:

Male Female Transgender

6. What is the primary population you serve?
   - □ Inner city
   - □ Urban/suburban
   - □ Small town
   - □ Rural
   - □ Geographically remote
   - □ Cannot identify a primary population
   - □ Other, please specify: ______________________________

7. What category describes your main patient care setting? (Pick only one)
   - □ Private (excluding freestanding walk-in clinics)
   - □ Community clinic/health centre
   - □ Free-standing walk-in clinic
   - □ Academic health science centre
   - □ Community hospital
   - □ Nursing home
   - □ Other, please specify: ______________________________

8. How is your main patient care setting organized?
□ Solo
□ Group
□ Other, please specify: ______________________________

DATA / COMMUNICATION

9. Do you use Electronic Medical Records (EMR)?
Yes No (skip to Question 10)

A. Does your EMR have a standard referral template that you use?
Yes No (skip to Question 10)

B. How was the referral template developed?
□ Self-generated
□ EMR vendors
□ Other General Practitioners/Family Physicians
□ Regional Health Authority / Local Health Information Network
□ Template from Otolaryngologist
□ Elsewhere, please specify: ______________________________

10. Do you have an Otolaryngology specific referral template?
Yes No

11. Which communication channel do you use when making referral requests? (Check all that apply)
□ Email
□ Fax
□ Mailed Letter
□ Telephone
□ Other, please specify: ______________________________

REFERRAL PROCESS

Please indicate your level of agreement with the following statements: (check)

	Strongly Agree	Agree	Neutral	Disagree	Strongly Disagree
12. When referring a patient, I provide the information required by the specialist to prioritize the patient.					
13. I do not order any additional tests (e.g.: bloodwork, diagnostic imaging) because specialists repeat the workup.					
14. Regulations that prevent General Practitioners/Family Physicians from ordering advanced diagnostic tests create inefficiencies in the referral process.					
15. I prefer to refer patients to Otolaryngologists that I know.					
16. I refer to specific Otolaryngologists based on the sub-specialty involved (example: otology, head and neck cancer, rhinology).					
17. I refer to specific Otolaryngologists based on word-of-mouth from patients and colleagues.					
18. I base my decision to refer to specific Otolaryngologists on the individual's wait time.					
19. It is challenging to know who sees what kind of specific problems in Otolaryngology.					
20. I would prefer to have a central referral system from which referrals are distributed to the appropriate sub-specialist in Otolaryngology.					

21. If a specialist does NOT see a particular problem, I would prefer that:
(Check all that apply)

_______ The referral is sent back to my office
_______ A different specialist is recommended
_______ The referral is forwarded directly to another specialist who sees the problem with notification to my office
Other:____

CONSULTATION PROCESS FEEDBACK

22. I prefer to receive notes from Otolaryngology in the following settings: (Circle all that apply).

Initial Consultation / Change in Management / Operative Note / Every Encounter

Please indicate your level of agreement with the following statements: (check)

	Strongly Agree	Agree	Neutral	Disagree	Strongly Disagree
23. I prefer to be informed when the Otolaryngologist has arranged an appointment with my patient.					
24. I am happy with the current wait times for my patients to be seen by Otolaryngology.					
25. After consultation, I receive communication from the specialist within an acceptable timeframe.					
26. I prefer for the Otolaryngologist to remain highly involved in the care of my patient rather than make a number of recommendations for me to follow.					
27. Overall, I am satisfied with the <u>referral</u> process I have had to date with Otolaryngology in London.					
28. Overall, I am satisfied with the <u>consultation</u> service I have had to date with Otolaryngology in London.					

GENERAL COMMENTS

30. Comments to make the referral process better: ______________________________

31. Comments to make the consultation process better: ______________________________

32. Other comments: ______________________________

Thank you very much for your participation!
